# Regulation of Root Exudation in Wheat Plants in Response to Alkali Stress

**DOI:** 10.3390/plants13091227

**Published:** 2024-04-28

**Authors:** Huan Wang, Shuting Zhao, Zexin Qi, Changgang Yang, Dan Ding, Binbin Xiao, Shihong Wang, Chunwu Yang

**Affiliations:** 1 Department of Agronomy, Jilin Agricultural University, Changchun 130118, China; angelfuture@163.com (H.W.); 20232052@mails.jlau.edu.cn (S.Z.); 20232053@mails.jlau.edu.cn (Z.Q.); aubunuri@163.com (D.D.); 2 Wheat Research Institute, Gansu Academy of Agricultural Sciences, Lanzhou 730070, China; cgyang1985@126.com; 3 Key Laboratory of Molecular Epigenetics of Ministry of Education, Northeast Normal University, Changchun 130024, China; xiaobb663@nenu.edu.cn (B.X.); yangcw809@nenu.edu.cn (C.Y.)

**Keywords:** wheat, pH regulation, root secretion, respiration, carboxylic acid

## Abstract

Soil alkalization is an important environmental factor limiting crop production. Despite the importance of root secretion in the response of plants to alkali stress, the regulatory mechanism is unclear. In this study, we applied a widely targeted metabolomics approach using a local MS/MS data library constructed with authentic standards to identify and quantify root exudates of wheat under salt and alkali stresses. The regulatory mechanism of root secretion in alkali-stressed wheat plants was analyzed by determining transcriptional and metabolic responses. Our primary focus was alkali stress-induced secreted metabolites (AISMs) that showed a higher secretion rate in alkali-stressed plants than in control and salt-stressed plants. This secretion was mainly induced by high-pH stress. We discovered 55 AISMs containing –COOH groups, including 23 fatty acids, 4 amino acids, 1 amino acid derivative, 7 dipeptides, 5 organic acids, 9 phenolic acids, and 6 others. In the roots, we also discovered 29 metabolites with higher levels under alkali stress than under control and salt stress conditions, including 2 fatty acids, 3 amino acid derivatives, 1 dipeptide, 2 organic acids, and 11 phenolic acids. These alkali stress-induced accumulated carboxylic acids may support continuous root secretion during the response of wheat plants to alkali stress. In the roots, RNAseq analysis indicated that 5 6-phosphofructokinase (glycolysis rate-limiting enzyme) genes, 16 key fatty acid synthesis genes, and 122 phenolic acid synthesis genes have higher expression levels under alkali stress than under control and salt stress conditions. We propose that the secretion of multiple types of metabolites with a –COOH group is an important pH regulation strategy for alkali-stressed wheat plants. Enhanced glycolysis, fatty acid synthesis, and phenolic acid synthesis will provide more energy and substrates for root secretion during the response of wheat to alkali stress.

## 1. Introduction

As the ecological environment continues to deteriorate through unreasonable development and use, the global area of saline land has increased yearly [1,2,3,4,5]. The harmful salts in saline soils mainly include NaCl, Na_2_SO_4_, NaHCO_3_, and Na_2_CO_3_. About 46% of saline soils contain only neutral salts, NaCl, and Na_2_SO_4_, but the remaining 54% contain both neutral salts and alkaline salts [6]. The stress type exerted by NaCl and/or Na_2_SO_4_ is defined as salt stress, whereas the stress type exerted by NaHCO_3_ and/or Na_2_CO_3_ is defined as alkali stress [7,8]. Previous studies have verified that the destructive effect of alkaline salt stress on plants is significantly stronger than that of neutral salt stress at the same salinity [7,8,9]. Soil alkalization has caused serious environmental problems in some areas of the world. For example, in northeastern China, about 50% of grassland is threatened by soil alkalization [10]. Soil pH in the alkalized area even reaches above 10.5. Only a few alkali-resistant halophytes can survive under such heavily alkaline conditions, and no crop can survive extreme alkalinity. Therefore, further research on soil alkalization and alkali stress is warranted. 

Salt stress produces negative effects on plants through osmotic stress and ion toxicity. However, in addition to osmotic stress and ion toxicity, alkali stress produces high-pH stress. High pH caused by alkali stress can lead to the precipitation of Ca^2+^, Mg^2+^, Fe^2+^, Mn^2+^, Cu^2+^, Zn^2+^, and PO_4_^3−^ to surrounding roots, which induces a reduction in the bioavailability of nutrient elements [2,9]. Additionally, a proton gradient across root plasma membranes is the driving force for mineral ion uptake. HCO_3_^−^ or CO_3_^2−^ from alkaline soils will neutralize the proton outside the root plasma membrane, thus breaking the proton gradient and inhibiting the uptake of mineral ions. The plants living in alkaline soils must regulate rhizosphere pH to alleviate nutrient stress. Therefore, the pH regulation of the roots is essential for alkali tolerance in plants.

In the past 30 years, great progress has been made in several areas of salt stress study, such as ion homeostasis, signal transduction, and hormone regulation [11,12,13,14,15]. To date, multilevel signal networks mediating salt tolerance and Na^+^ compartmentalization mechanisms at the subcellular level have been elucidated [11,12,13,14,15,16]. However, relatively few studies have focused on plant alkali tolerance [3,4,17,18,19,20,21,22,23,24,25,26,27,28]. Important progress in research on plant alkali tolerance has been made in Arabidopsis [21], maize [25], and wheat [27], in which H^+^-ATPase was demonstrated to play an important role in alkali tolerance.

Our group and other researchers have found that root secretion is the main pH regulation pathway of plants under alkali stress [29,30,31]. Root exudates usually include amino acids, phenolics, fatty acids, organic acids, and carbohydrates [22,32]. Secretion of organic acids induced by alkali stress has been reported in many plants, such as *P. tenuiflora* [30,33], grape plants [31], and *Chloris virgata* [29]. However, the physiological and molecular mechanisms underlying root secretion regulation during the response of plants to alkali stress are poorly understood. Wheat provides about 20% of the calories consumed by humans [34]. Soil alkalization is an important factor limiting wheat production in northern China. To explore the specific effects of high pH caused by alkali stress on root secretion, we applied salt stress and alkali stress treatments at the same Na^+^ concentration and total salt concentration but with different pH values. Thus, differences in plant root secretion in response to the two stress conditions were mainly attributed to pH differences. In this study, we identified and quantified root exudates of wheat under salt and alkali stresses. To ascertain the regulatory mechanism of root secretion in wheat under alkali stress, we also analyzed the transcriptional and metabolic responses of wheat roots to alkali stress.

## 2. Results

### 2.1. Components of Root Exudates

**Figure 1:**
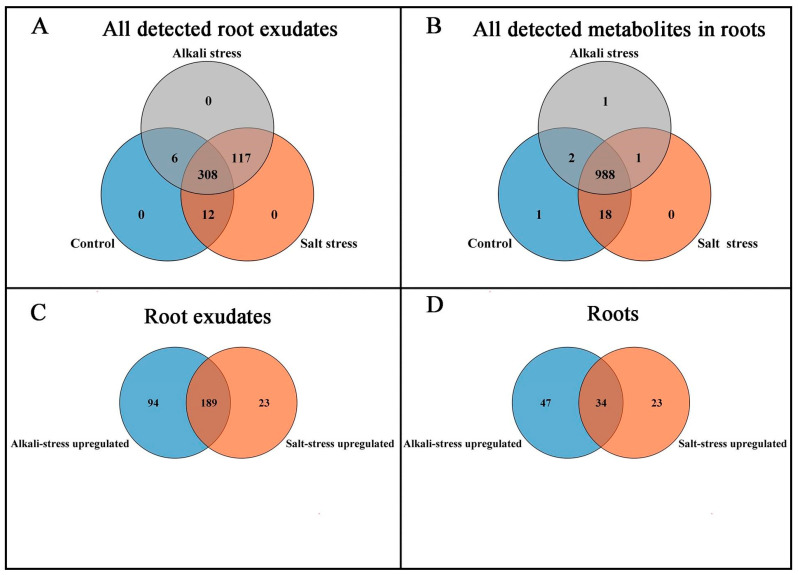
Comparison of metabolite components in root exudates and roots of wheat plants under control, salt stress, and alkali stress conditions. (**A**) Number of all detected root exudates; (**B**) number of all detected metabolites in roots; (**C**) number of the metabolites with enhanced root secretion rate; (**D**) number of the metabolites with upregulated accumulation in roots. The 30-day-old wheat seedlings were treated with salt (88 mM Na^+^ and pH 6.7) and alkali (88 mM Na^+^ and pH 8.8) solutions for 3 days. Three biological replicates were used for each treatment.

**Figure 2:**
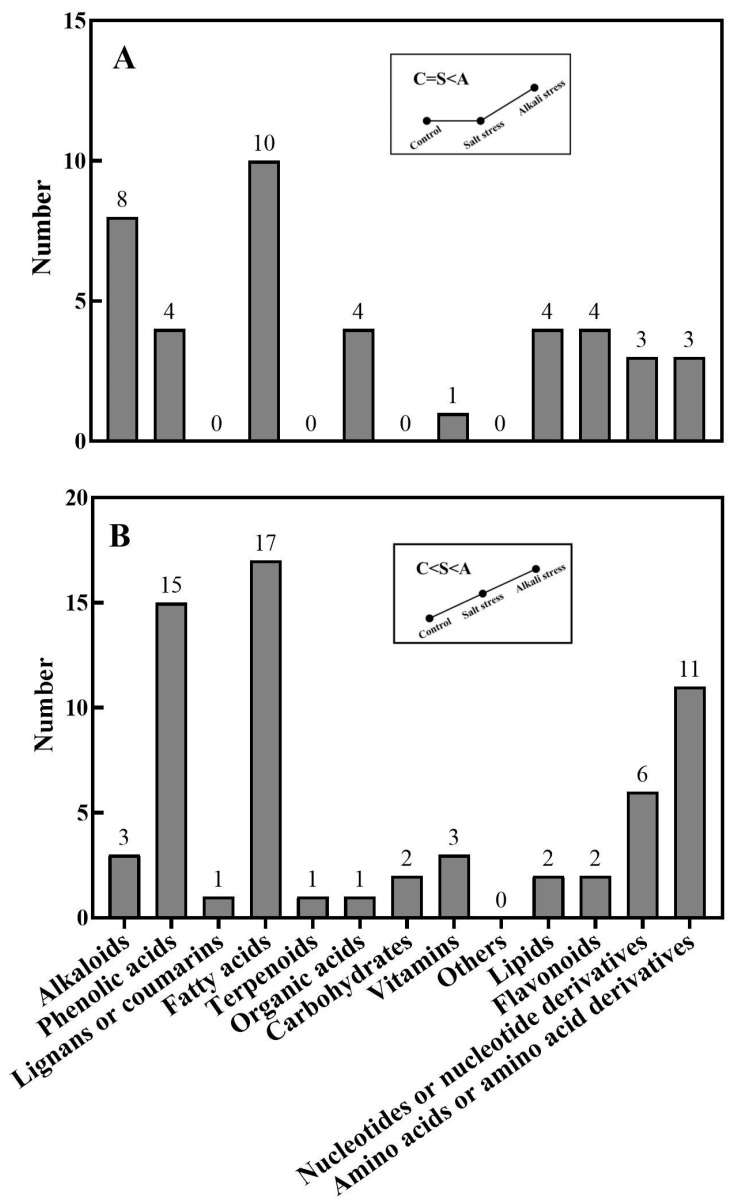
Alkali stress-induced secreted metabolites. The number of metabolites for each type of metabolite was displayed. (**A**) Control and salt-stressed plants showed a similar secretion rate for each metabolite, with a lower secretion rate than that in alkali-stressed plants; (**B**) alkali-stressed plants > salt-stressed plants > control plants in the secretion rate of metabolites. The 30-day-old wheat seedlings were treated with salt (88 mM Na^+^ and pH 6.7) and alkali (88 mM Na^+^ and pH 8.8) solutions for 3 days. Three biological replicates were used for each treatment.

**Figure 3:**
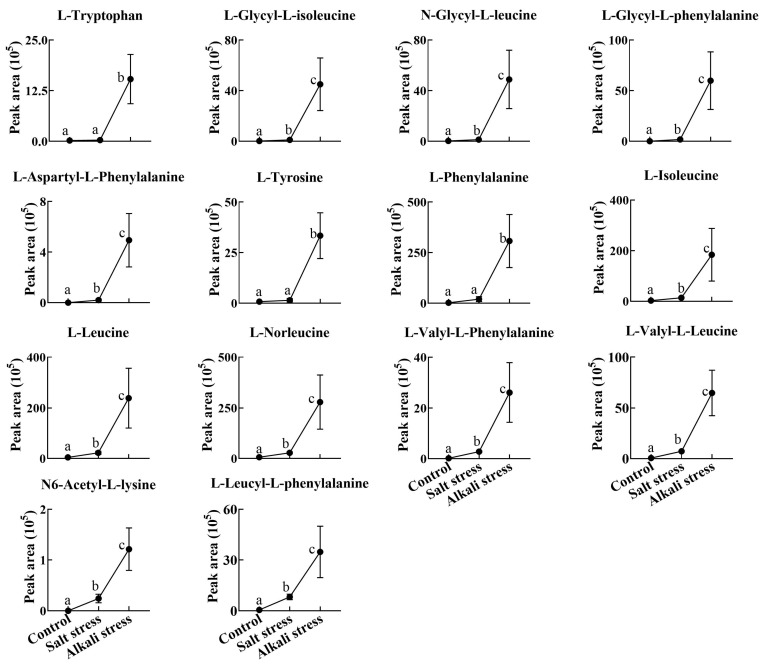
Comparative effects of salt and alkali stresses on the secretion of amino acids and amino acid derivatives in wheat plants. Alkali stress-induced secreted amino acids or amino acid derivatives are displayed. The 30-day-old wheat seedlings were treated with salt stress (88 mM Na^+^ and pH 6.7) and alkali stress (88 mM Na^+^ and pH 8.8) solutions for 3 days. Three biological replicates were used for each treatment. Different letters above the bar indicate significant differences.

**Figure 4:**
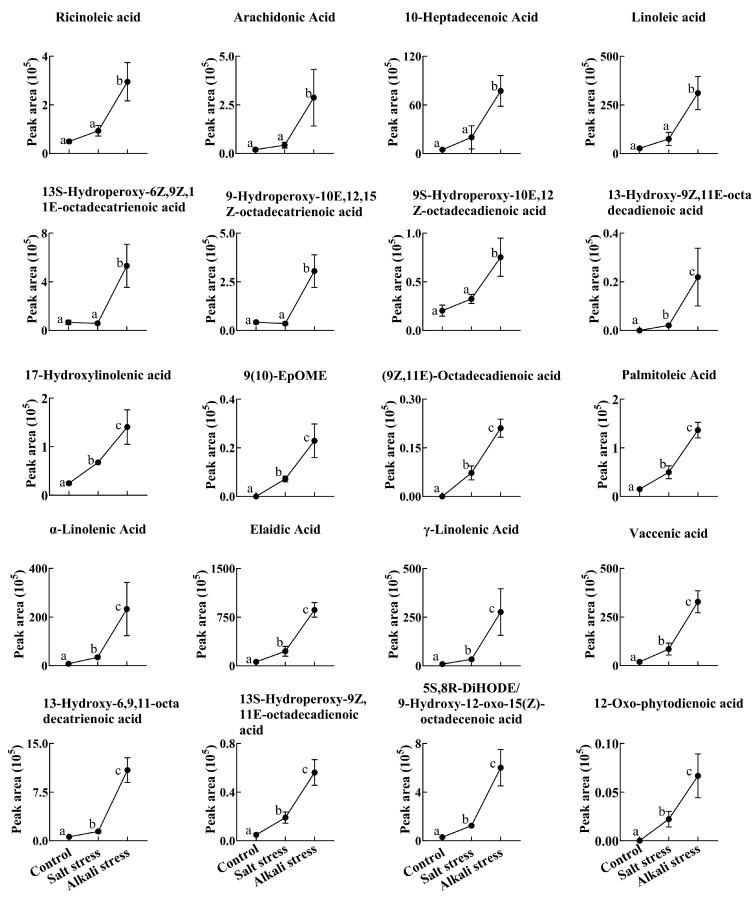
Comparative effects of salt and alkali stresses on the secretion of unsaturated fatty acids in wheat plants. Alkali stress-induced secreted unsaturated fatty acids are displayed. The 30-day-old wheat seedlings were treated with salt (88 mM Na^+^ and pH 6.7) and alkali (88 mM Na^+^ and pH 8.8) solutions for 3 days. Three biological replicates were used for each treatment. Different letters above the bar indicate significant differences.

**Figure 5:**
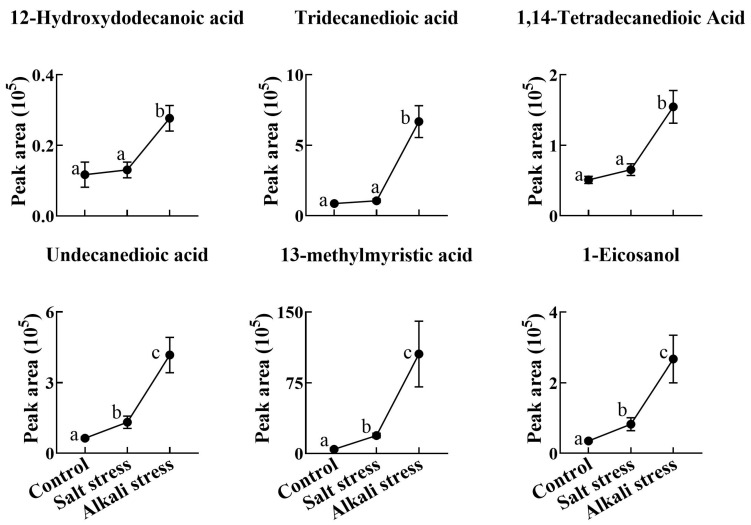
Comparative effects of salt and alkali stresses on the secretion of saturated fatty acids in wheat plants. Alkali stress-induced secreted saturated fatty acids are displayed. The 30-day-old wheat seedlings were treated with salt stress (88 mM Na^+^ and pH 6.7) and alkali stress (88 mM Na^+^ and pH 8.8) solutions for 3 days. Three biological replicates were used for each treatment. Different letters above the bar indicate significant differences.

**Figure 6:**
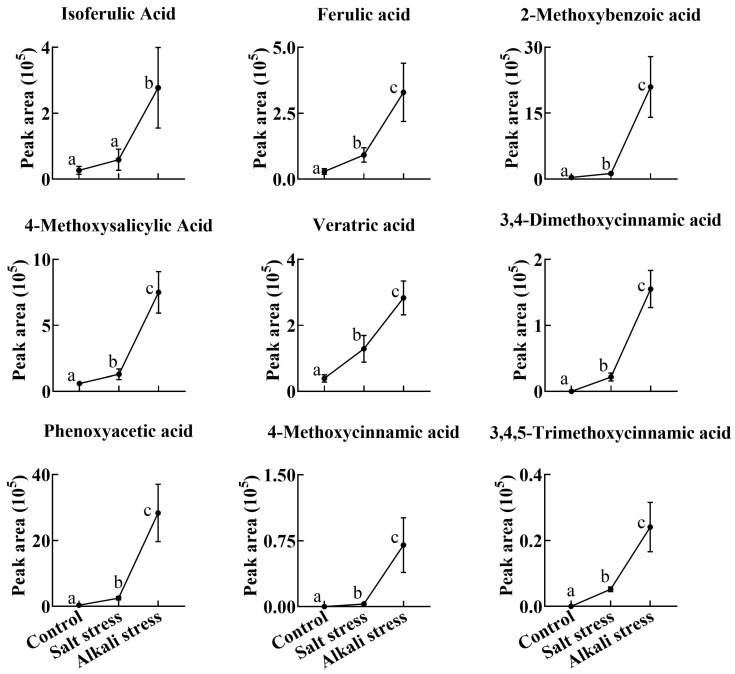
Comparative effects of salt and alkali stresses on the secretion of phenolic acids with a –COOH group in wheat plants. Alkali stress-induced secreted phenolic acids are displayed. The 30-day-old wheat seedlings were treated with salt stress (88 mM Na^+^ and pH 6.7) and alkali stress (88 mM Na^+^ and pH 8.8) solutions for 3 days. Three biological replicates were used for each treatment. Different letters above the bar indicate significant differences.

**Figure 7:**
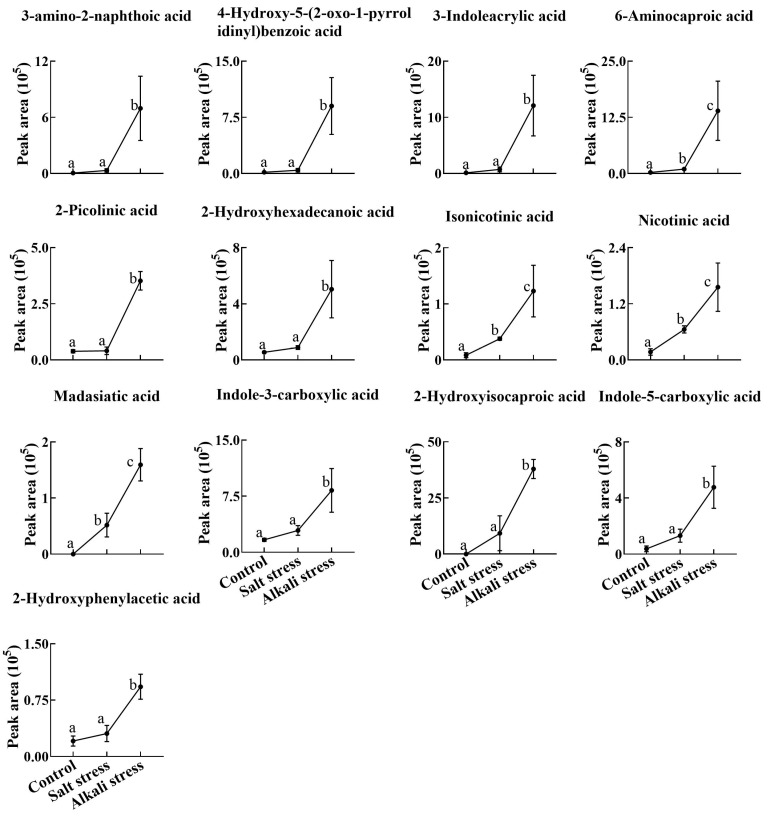
Comparative effects of salt and alkali stresses on the secretion of other carboxylic acids in wheat plants. The 30-day-old wheat seedlings were treated with salt stress (88 mM Na^+^ and pH 6.7) and alkali stress (88 mM Na^+^ and pH 8.8) solutions for 3 days. Three biological replicates were used for each treatment. Different letters above the bar indicate significant differences.

We used a high throughput metabolomic method to detect metabolites in the root exudates (Figure 1A) and root tissues of wheat plants (Figure 1B). Collectively, we detected 443 root exudates in wheat plants under three conditions (Figure 1A), including 75 fatty acids, 52 lipids, 27 organic acids, 31 amino acids or amino acid derivatives, 81 phenolic acids, 28 nucleotides or nucleotide derivatives, 54 flavonoids, 38 alkaloids, 7 terpenoids, 18 carbohydrates, 8 vitamins, 7 lignans or coumarins, and 17 others (Table S1). In wheat plants, 326 root exudates were detected under control conditions, 437 under salt stress, and 431 under alkali stress (Table S1 and Figure 1A). We particularly focused on alkali stress-induced secreted metabolites (AISMs), which were found at a higher root secretion rate under alkali stress condition than under control and salt stress conditions. The number of AISMs for each type of metabolite is displayed in Figure 2A,B. In Figure 2A, salt stress did not affect the secretion rate of the metabolites, but alkali stress enhanced the secretion rate. Conversely, in Figure 2B, both salt stress and alkali stress enhanced the secretion rate of the metabolites, with greater enhancement in alkali stress than in salt stress. We discovered 105 AISMs in wheat root exudates, including 27 fatty acids, 6 amino acids, 1 amino acid derivative, 7 dipeptides, 5 organic acids, 19 phenolic acids, 9 nucleotides or nucleotide derivatives, 6 flavonoids, 1 lignan or coumarin, 11 alkaloids, 2 carbohydrates, 1 terpenoid, 6 lipids, and 4 vitamins (Figure 1C and Figure 2). Of 105 AISMs, 55 AISMs contained the –COOH group, including 23 fatty acids, 4 amino acids, 1 amino acid derivative, 7 dipeptides, 5 organic acids, 9 phenolic acids, 3 alkaloids, 1 terpenoid, and 2 others (Figure 3, Figure 4, Figure 5, Figure 6 and Figure 7). These data revealed that fatty acids, amino acids, dipeptides, and phenolic acids were dominant AISMs for alkali-stressed wheat plants. Some plant “hub” fatty acids, such as γ-linolenic acid, arachidonic acid, α-linolenic acid, linoleic acid, and palmitoleic acid also showed higher root secretion rates under alkali stress conditions than under control and salt stress conditions (Figure 4 and Figure 5). All of the three aromatic amino acids (tryptophan, tyrosine, and phenylalanine) were discovered in the list of AISMs (Figure 3).

### 2.2. Metabolic Profiling of the Roots

**Figure 8:**
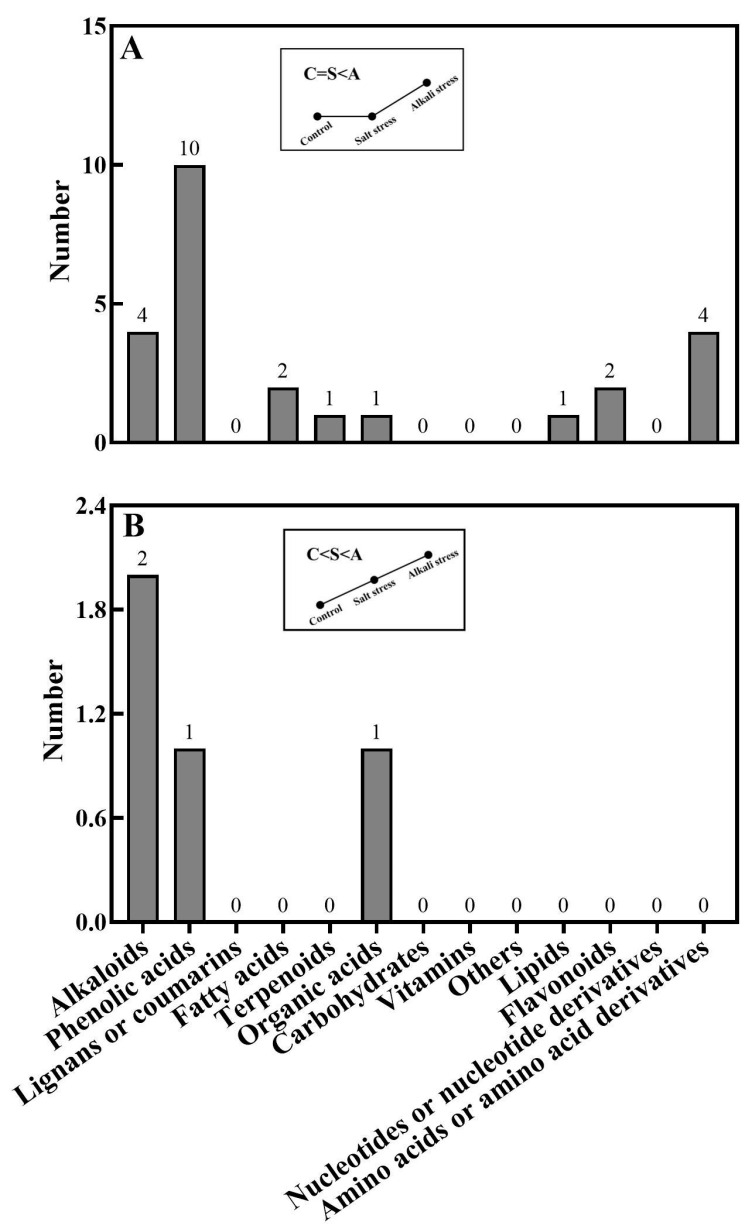
Alkali stress-induced accumulated metabolites in wheat roots. The number of metabolites in each type is displayed. (**A**) Control and salt-stressed plants showed similar levels for each metabolite, with lower levels than those in alkali-stressed plants; (**B**) alkali-stressed plants > salt-stressed plants > control plants in levels of metabolites. The 30-day-old wheat seedlings were treated with salt stress (88 mM Na^+^ and pH 6.7) and alkali stress (88 mM Na^+^ and pH 8.8) solutions for 3 days. Three biological replicates were used for each treatment.

In wheat roots, we collectively detected 1011 metabolites, including 91 fatty acids, 93 lipids, 81 organic acids, 97 amino acids or amino acid derivatives, 164 phenolic acids, 71 nucleotides or nucleotide derivatives, 128 flavonoids, 111 alkaloids, 22 terpenoids, 64 carbohydrates, 16 vitamins, 44 lignans or coumarins, 5 quinones, and 24 others (Figure 1B,D and Table S2). Of these metabolites, 106 metabolites displayed different concentrations under control and salt stress conditions, 224 metabolites displayed different concentrations under control and alkali stress conditions, and 144 metabolites were differentially accumulated under salt stress and alkali stress conditions. We displayed alkali stress-induced accumulated metabolites (AIAMs), which were found at a higher concentration in the roots under alkali stress conditions than under control and salt stress conditions (Figure 8). The number of AIAMs for each type of metabolite is shown in Figure 8A,B. In Figure 8A, salt stress did not affect the accumulation of the metabolites, but alkali stress enhanced the accumulation. In Figure 8B, both salt stress and alkali stress enhanced the concentration of the metabolites, with greater enhancement in alkali stress than in salt stress. We discovered 29 AIAMs in wheat roots, including 2 fatty acids (γ-linolenic acid and α-linolenic acid), 3 amino acid derivatives, 1 dipeptide, 2 organic acids (shikimic acid and muconic acid), 11 phenolic acids, 2 flavonoids, 1 lipid, 1 terpenoid, and 6 alkaloids (Figure 8 and Table S3). Integrated analysis of root exudates and root metabolome data showed higher levels of γ-linolenic acid and α-linolenic acid in alkali-stressed roots than in control and salt-stressed roots, as well as a faster secretion rate in alkali-stressed roots than in control and salt-stressed roots.

### 2.3. Gene Expression Response in the Roots

The results of the RNAseq were validated with real-time quantitative PCR (qRT-PCR) (Table S4). In 10 of the 12 randomly selected genes, the fold changes of the RNAseq experiment were similar to those of the qRT-PCR experiment, indicating that the results of the RNAseq experiment were reliable (Table S4). Compared with the control, salt stress upregulated the expression of 2108 genes and downregulated the expression of 1470 genes, whereas alkali stress upregulated the expression of 8542 genes and downregulated the expression of 6764 genes. The expression level of 5967 genes was higher in alkali-stressed roots than in salt-stressed roots, and 8147 genes displayed a lower level of expression in alkali-stressed roots than in salt-stressed roots. Alkali stress-induced genes (AIGs) were considered those with an expression level higher in alkali-stressed plants than in control and salt-stressed plants. We discovered 5764 AIGs, which were exposed to KEGG enrichment. The AIGs were enriched in phenylpropanoid biosynthesis, amino acid metabolism, nitrogen metabolism, amino acid-related enzymes, phenylalanine metabolism, flavonoid biosynthesis, alpha-linolenic acid metabolism, and other pathways (Table S5). AIGs involved in alkali tolerance are shown in Figures S1–S6. The AIGs included 18 NRT1/PTR FAMILY (*NPF*) genes, 22 *NRT* genes (Figure S1), 11 1-aminocyclopropane-1-carboxylate (ACC) oxidase genes, and 38 ethylene-responsive transcription factor genes (Figure S2). In the AIG list, we also discovered 29 glycolysis/gluconeogenesis genes including 5 glycolysis rate-limiting enzyme (6-phosphofructokinase) genes, and 16 key fatty acid synthesis genes (4 *FabG* genes, 1 *FabF* gene, 1 medium-chain acyl-[acyl-carrier-protein] hydrolase gene and 4 long-chain acyl-CoA synthetase genes) (Figure S3). Additionally, we also found 122 phenolic acid synthesis genes in the list of AIGs (Table S5), including 4 phenylalanine ammonia-lyase (*PAL*, phenolic acid synthesis rate-limiting enzyme) genes, 7 4-coumarate-CoA ligase (*4CL*) genes, and 2 trans-cinnamate 4-monooxygenase genes (Figure S4). The expression level of 22 peptide transporter genes, 3 oligopeptide transporter genes, 6 protease genes, 1 ubiquitin-conjugating enzyme gene, and 13 E3 ubiquitin-protein ligase genes was also higher in alkali-stressed roots than in control and salt-stressed roots (Figures S5 and S6).

## 3. Discussion

Root secretion has a vital role in the tolerance of plants to abiotic stresses, such as phosphorus deficiency, heavy metal pollution, aluminum toxicity, and alkali stress [22,32]. The roles of organic acid secretion in pH regulation under alkali stress have been reported in grapevine roots [35], grape plants [31], and *C. virgata* plants [29]. High pH caused by alkali stress can precipitate various mineral element ions at the rhizosphere, leading to nutrient deficiency [28]. High pH can also induce the over-accumulation of Na^+^ and enhance ion toxicity [28]. Thus, the regulation of pH at the rhizosphere or within roots is vital for plant survival under high alkali conditions. In this study, we detected a diverse array of metabolites covering most types of metabolites in the root exudates of alkali-stressed wheat plants. We particularly focused on secreted metabolites induced by alkali stress (high-pH). We discovered 55 AISMs contained a –COOH group, including 23 fatty acids, 4 amino acids, 1 amino acid derivative, 7 dipeptides, 5 organic acids, 9 phenolic acids, 3 alkaloids, 1 terpenoid, and 2 others. We propose that the secretion of multiple types of metabolites with the –COOH group may be an important pH regulation strategy for wheat roots under alkali stress. Recently, root exudates of a halophyte *Puccinellia tenuiflora* under alkali stress were also analyzed by a metabolomics approach [33]. In *P. tenuiflora* plants, 75 AISMs with the –COOH group were discovered, including 42 fatty acids, 3 amino acid derivatives, 22 phenolic acids, and 8 organic acids [33]. Our recently published work revealed that halophyte *Leymus chinensis* responded to alkali stress via the secretion of phenolic acids, free fatty acids, organic acids, and amino acids [36]. However, that study did not apply salt stress treatment, so the root secretion response of *L. chinensis* to a high pH was not explored. The above data demonstrated that the secretion of fatty acids, phenolic acids, and organic acids was the common response of plants to alkali stress. However, amino acids and dipeptides were discovered in AISMs of wheat and not in *P. tenuiflora*. This suggests that wheat and the halophyte *P. tenuiflora* have different pH regulation strategies under alkali stress. The secretion of amino acids and dipeptides may play more important roles in wheat alkali tolerance.

Glycolysis provides the reducing power (ATP and NADH) and carbon source for metabolisms and the root secretion process. In the wheat roots, five 6-phosphofructokinase (glycolysis rate-limiting enzyme) genes displayed higher expression levels under alkali stress than under control and salt stress conditions (Figure S3). Enhanced glycolysis will provide more reducing power and carbon sources for the synthesis of fatty acids, phenolic acids, and organic acids to support their secretion into the rhizosphere during the response of wheat to alkali stress. We also focused on metabolites with a higher level in alkali-stressed wheat roots than in control and salt-stressed wheat roots, including 2 fatty acids, 3 amino acid derivatives, 1 dipeptide, 2 organic acids, 11 phenolic acids, 2 flavonoids, 1 lipid, 1 terpenoid, and 6 alkaloids. These alkali stress-induced accumulated carboxylic acids not only have roles in osmotic regulation but also directly or indirectly support root secretion during the response of wheat to alkali stress. The enhanced accumulation of carboxylic acids (e.g., amino acids, fatty acids, and organic acids) has also been observed in alkali-stressed rice [19], alfalfa [9], and sunflower [37]. RNAseq analysis showed that 16 key fatty acid synthesis genes and 122 phenolic acid synthesis genes (including rate-limiting enzyme genes *PAL*) have a higher expression level in wheat roots under alkali stress conditions than under control and salt stress conditions (Figure S4), indicating a strategy for the regulation of gene expression for the accumulation and secretion of fatty acids and phenolic acids during the response of wheat roots to alkali stress. Additionally, the expression level of 18 *NPF* genes and 25 peptide transporter genes was higher in alkali-stressed wheat roots than in control and salt-stressed wheat roots (Figure S1). The NPF family can transport multiple substrates, including chloride, potassium, carboxylate, plant hormones, peptides, nitrate, and metabolites containing a –COOH group [38]. The upregulated expression of the *NPF* genes may accelerate the secretion of metabolites containing the –COOH group and facilitate rhizosphere pH regulation in alkali-stressed wheat. Although we have identified some candidate genes that can mediate root secretion of wheat plants under alkali stress, some important questions remain, such as which genes mediate the co-expression of 19 *NPF* genes and 25 peptide transporter genes under alkali stress and what mechanism coordinates the production and secretion of AISMs. In wheat plants, alkali stress-induced secreted dipeptides and amino acids may be produced from protein degradation, while other secreted carboxylic acids may be generated from continuous biosynthesis. The upregulation of E3 ubiquitin-protein ligase genes, ubiquitin-conjugating enzyme genes, and protease genes facilitates the protein degradation that generates oligopeptides or amino acids (Figure S6), which provides materials for the secretion of dipeptides and amino acids by wheat roots under alkali stress. In wheat roots, the expression of 11 key ethylene synthesis genes and 38 ethylene-responsive transcription factor genes was particularly upregulated under alkali stress condition, suggesting that ethylene may mediate the response of wheat roots to alkali stress. It has been reported that ethylene plays a beneficial role in enhancing the salt tolerance of plants [39]. Ethylene may exert important effects in mediating the production and secretion of carboxylic acids and dipeptides during the response of wheat roots to alkali stress, which warrants further investigations.

## 4. Materials and Methods

### 4.1. Stress Treatment and Root Exudate Collection

Xiaobingmai33, a spring wheat variety widely cultivated in Northeast China, was selected as the test organism. The wheat seeds were provided by Prof. Jinsong Pang from Northeast Normal University, China. The seeds were sown in plastic pots containing sand. All pots (15 seedlings per pot; pot size height 19 cm and diameter 18.5 cm) were watered with half-strength Hoagland nutrient solution for 30 days in a greenhouse (23–25 °C day and 17–20 °C night, 16 h light). The experiment was conducted from mid-April to mid-May in Changchun, China. Based on the pH and salinity levels of moderate soda salt-alkaline in Northeast China, NaHCO_3_ and Na_2_CO_3_ were added at a 9:1 molar ratio (80 mM total salt concentration, 88 mM Na^+^ concentration, and pH 8.8) to mimic alkali stress conditions in the moderate soda salt–alkaline soil. To explore the specific effects of high-pH, NaCl and Na_2_SO_4_ were added at a 9:1 molar ratio (80 mM total salt concentration, 88 mM Na^+^ concentration, and pH 6.7) for the salt stress treatment. The control was cultured with a half-strength Hoagland nutrient solution (pH 6.6). The final pH values of the salt stress and alkali stress treatment solutions were determined after adding the nutrient solution. Wheat plants can finish their life cycle under such stress conditions. The pots with uniform wheat seedlings were treated with salt or alkali treatment solution containing nutrient components for three days, and then root exudates were collected and stored at −80 °C according to a method by Li et al. [33]. After root exudate collection, the root samples were collected and freeze-dried, and RNA samples were collected and stored at −80 °C. Ten plants were pooled as a biological replicate, with three biological replicates for metabolome analysis and RNA sequencing.

### 4.2. Metabolome Analysis

Metabolites in root exudates and root tissues were qualified and quantified using a widely targeted metabolomics approach based on a local MS-MS data library constructed with authentic standards [40]. The secretion rate of each metabolite was expressed as the relative amount (peak area) of g^−1^ root DW. Metabolites in root exudates and root tissues were measured according to Li et al. [33]. Briefly, freeze-dried root samples and freeze-dried root exudates were treated with 70% methanol, and then the extracts were loaded onto an LC–MS/MS system (QTRAP, AB SCIEX). A mixed sample of all extracts in equal volumes was loaded onto an LC–MS/MS system (QTRAP, AB SCIEX) to construct an MS2 spectral tag library. Retention time, *m*/*z* ratio, and fragmentation information were applied to identify each metabolite through an in-house database (MWDB, https://www.metware.cn accessed on 11 December 2021). All the metabolites identified were quantified using the MRM method [40]. We defined differentially accumulated metabolite (DAM) or differentially secreted metabolite (DSM) as VIP > 1, *p* value (*t* test) < 0.05, and |Log2(Fold change)| > 1.

### 4.3. RNAseq and qRT-PCR

Conventional methods were applied to conduct RNAseq experiments and data analyses [2]. Total RNA samples were used as input material for library construction. The prepared libraries were sequenced on an Illumina platform. Wheat reference genome and gene model annotation files were downloaded from the Ensembl Plants website (http://plants.ensembl.org/Triticum_aestivum/Info/Index accessed on 20 December 2021). The paired-end clean reads were mapped to the reference genome using Hisat2 v2.0.5. Differentially expressed genes (DEGs) were identified using the DESeq2 R package 1.20.0 (adjusted *p* value ≤ 0.05 and |log2fold change| ≥ 1) [41]. We applied the TBtools program to conduct GO and KEGG enrichments for DEGs [42]. The reliability of the RNAseq analysis was validated using qRT-PCR. *RLI*, *Actin 2*, *Actin 7*, and *β-tubulin* were selected as internal control genes. The expression level of the genes was calculated using the ΔΔCt method [43].

## 5. Conclusions

The secretion of multiple types of metabolites with a –COOH group is an important pH regulation strategy for alkali-stressed wheat plants. Enhanced glycolysis, fatty acid synthesis, and phenolic acid synthesis will provide more energy and substrates for root secretion during the response of wheat to alkali stress. In wheat plants, alkali stress-induced secreted dipeptides and amino acids may be produced from protein degradation, while other secreted carboxylic acids may be generated from continuous biosynthesis. Some *NPF* genes and peptide transporter genes may play important roles in the pH regulation of alkali-stressed wheat plants.

## Data Availability

All RNA sequencing raw data are deposited at NCBI (Accession number PRJNA970414). The datasets used and/or analyzed during the current study are available from the corresponding author upon reasonable request.

## Supplementary Materials

The following supporting information can be downloaded at: https://www.mdpi.com/article/10.3390/plants13091227/s1. Table S1: Mass spectrum information of all detected metabolites in root exudates of wheat plants. Table S2: Mass spectrum information of all detected metabolites in wheat roots. Table S3: Mass spectrum information of accumulated metabolites induced by alkali stress in wheat roots. Table S4: Results of qPCR. Table S5: KEGG enrichment for alkali stress-induced genes. Figure S1: Comparative effects of salt and alkali stresses on the expression of NPF genes. The 30-day-old wheat seedlings were treated with salt (88 mM Na^+^ and pH 6.7) and alkali (88 mM Na^+^ and pH 8.8) solutions for 3 days. Three biological replicates were used for each treatment. Different letters above the bars indicate significant differences. In the figures, each gene ID represents different members of a gene family. Figure S2: Comparative effects of salt and alkali stresses on the expression of genes involved in ethylene signal transduction. The 30-day-old wheat seedlings were treated with salt (88 mM Na^+^ and pH 6.7) and alkali (88 mM Na^+^ and pH 8.8) solutions for 3 days. Three biological replicates were used for each treatment. Different letters above the bars indicate significant differences. In the figures, each gene ID represents different members of a gene family. Figure S3: Comparative effects of salt and alkali stresses on the expression of the genes involved in glycolysis and fatty acid synthesis. The 30-day-old wheat seedlings were treated with salt (88 mM Na^+^ and pH 6.7) and alkali (88 mM Na^+^ and pH 8.8) solutions for 3 days. Each treatment had three biological replicates. Different letters above the bars indicate significant differences. In the figures, each gene ID represents different members of a gene family. Figure S4: Comparative effects of salt and alkali stresses on the expression of genes involved in phenolic acid synthesis. The 30-day-old wheat seedlings were treated with salt (88 mM Na^+^ and pH 6.7) and alkali (88 mM Na^+^ and pH 8.8) solutions for 3 days. Three biological replicates were used for each treatment. Different letters above the bars indicate significant differences. In the figures, each gene ID represents different members of a gene family. Figure S5: Comparative effects of salt and alkali stresses on the expression of peptide transporter genes. The 30-day-old wheat seedlings were treated with salt (88 mM Na^+^ and pH 6.7) and alkali (88 mM Na^+^ and pH 8.8) solutions for 3 days. Each treatment had three biological replicates. Different letters above the bars indicate significant differences. In the figures, each gene ID represents different members of a gene family. Figure S6: Comparative effects of salt and alkali stresses on the expression of the genes involved in protein degradation. The 30-day-old wheat seedlings were treated with salt (88 mM Na^+^ and pH 6.7) and alkali (88 mM Na^+^ and pH 8.8) solutions for 3 days. Three biological replicates were used for each treatment. Different letters above the bars indicate significant differences. In the figures, each gene ID represents different members of a gene family.
