# Supplementary figures and images for: Regulation of Root Exudation in Wheat Plants in Response to Alkali Stress
Source: Plants (Basel). 2024 Apr 28;13(9):1227. doi: 10.3390/plants13091227 (PMC11085862; doi:10.3390/plants13091227)

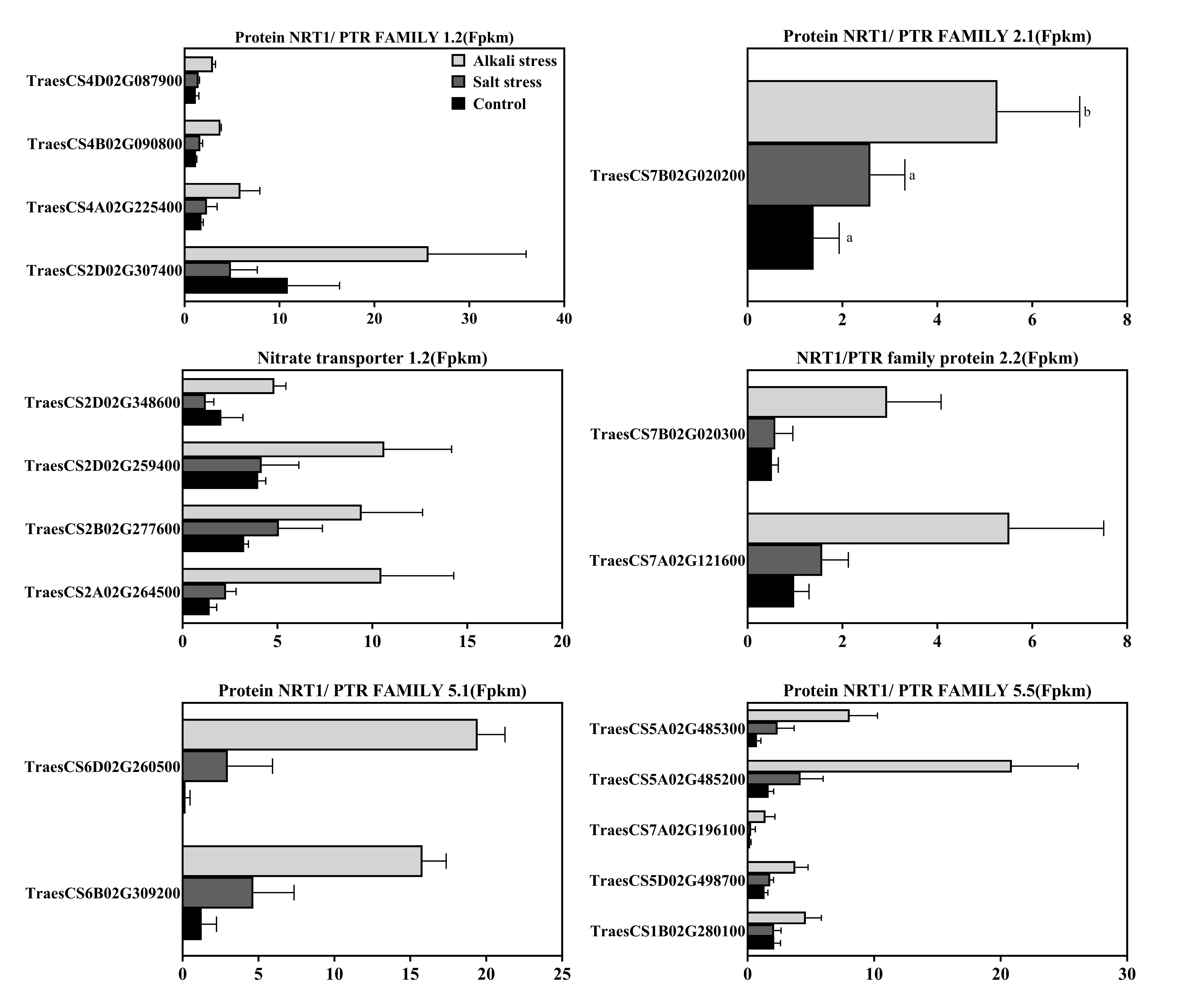

Supplement: Supplementary file 1 [file plants-13-01227-s001.zip › Figure S1.jpg]

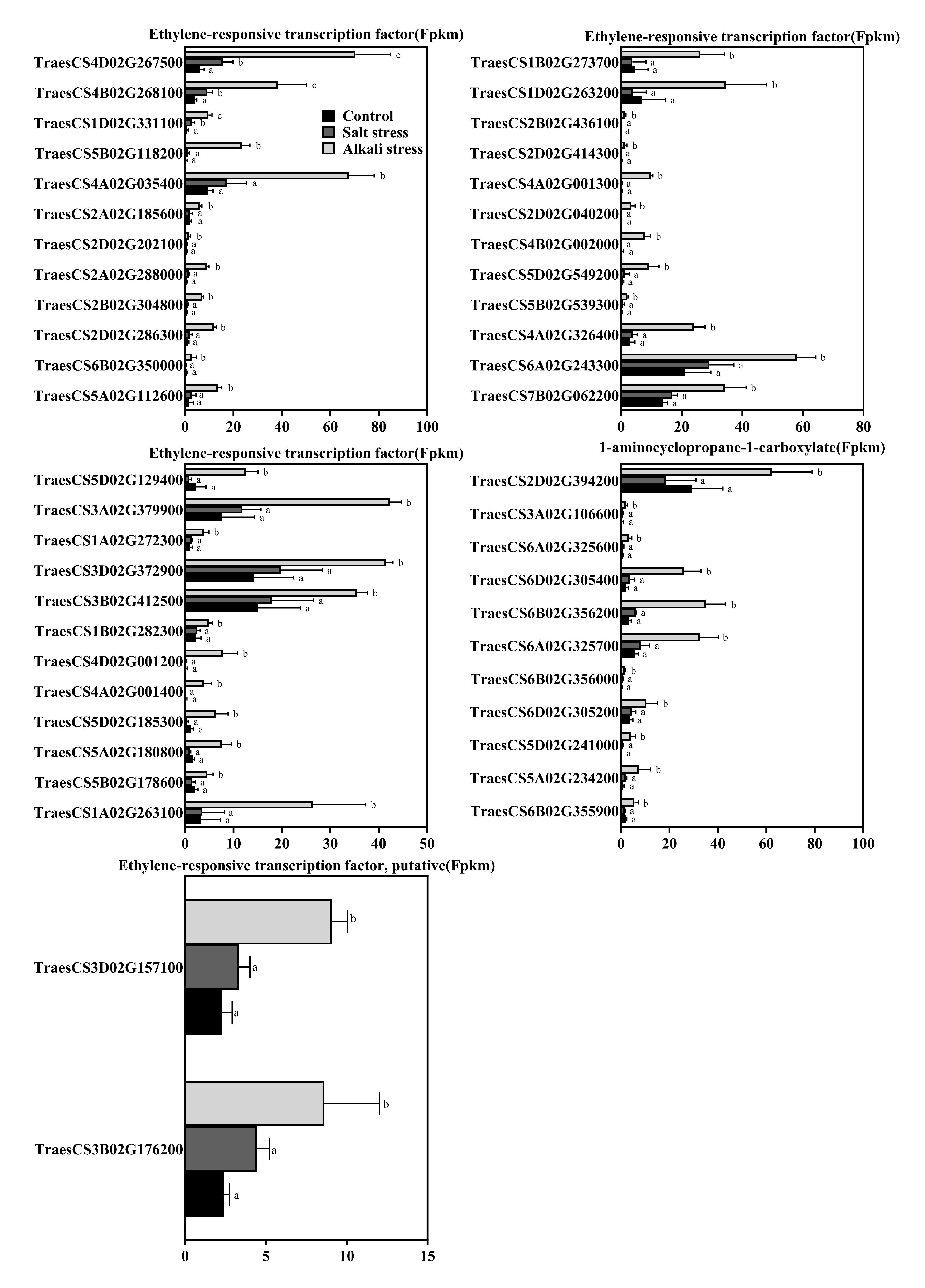

Supplement: Supplementary file 1 [file plants-13-01227-s001.zip › Figure S2.jpg]

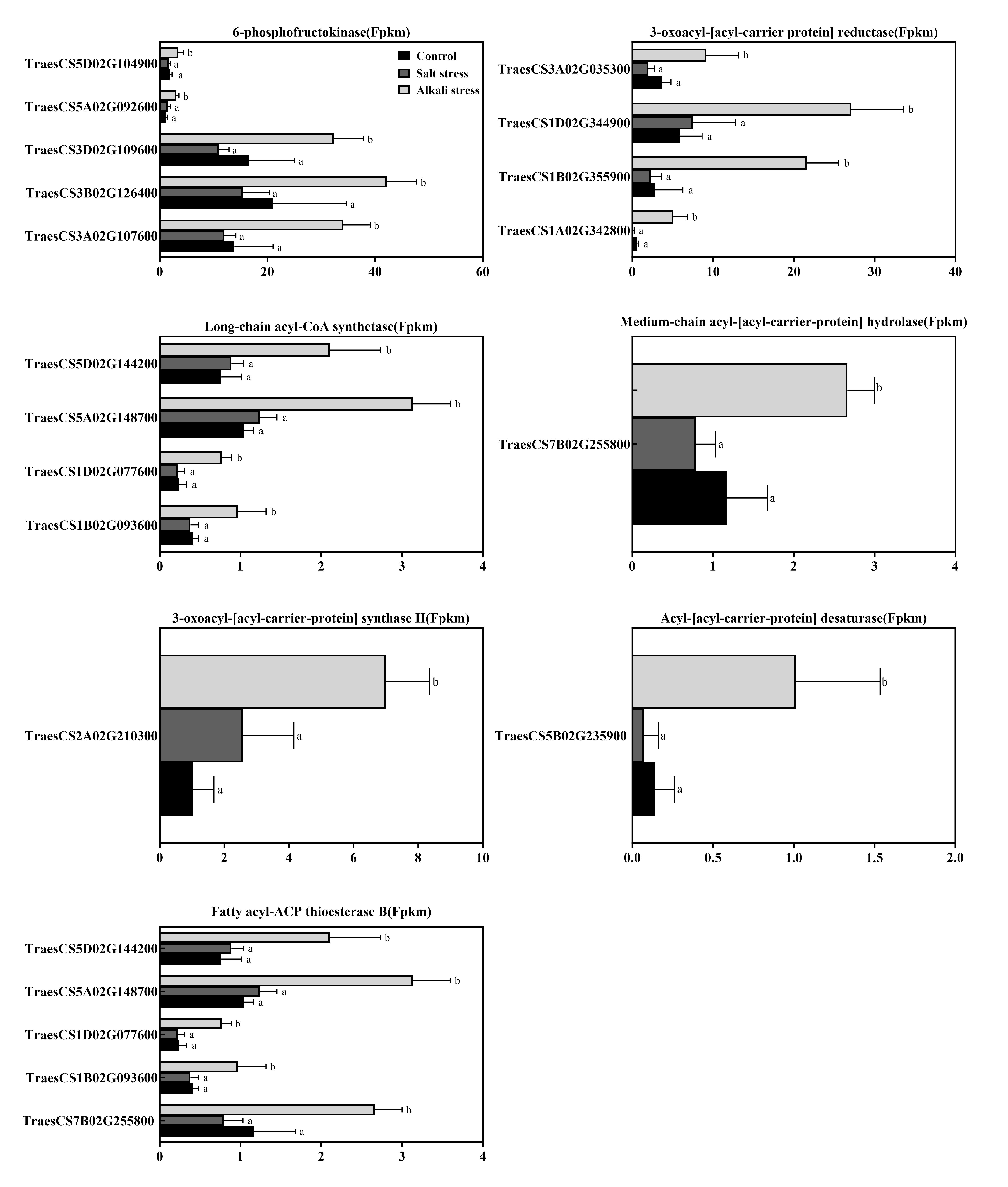

Supplement: Supplementary file 1 [file plants-13-01227-s001.zip › Figure S3.jpg]

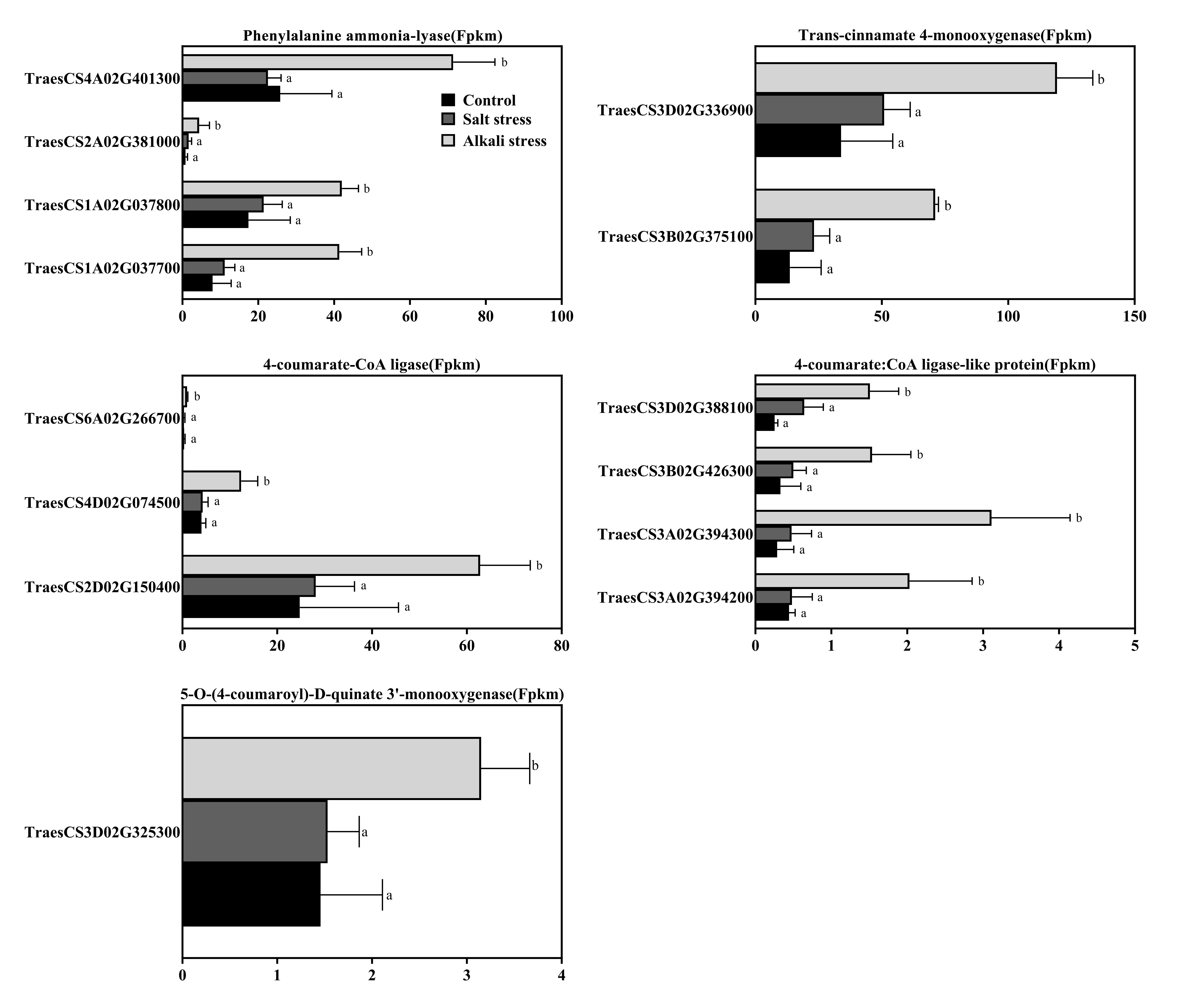

Supplement: Supplementary file 1 [file plants-13-01227-s001.zip › Figure S4.tif]

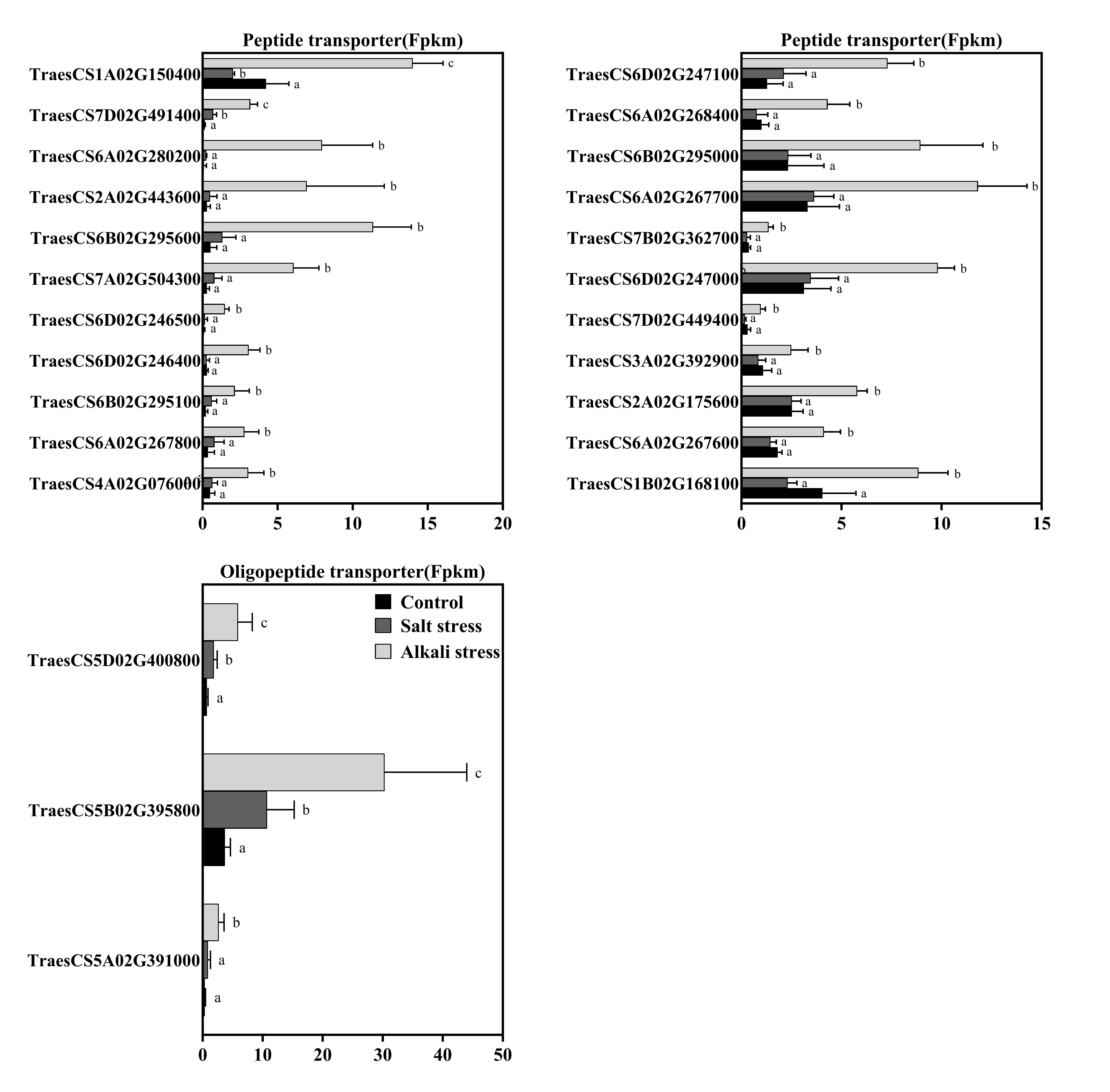

Supplement: Supplementary file 1 [file plants-13-01227-s001.zip › Figure S5.jpg]

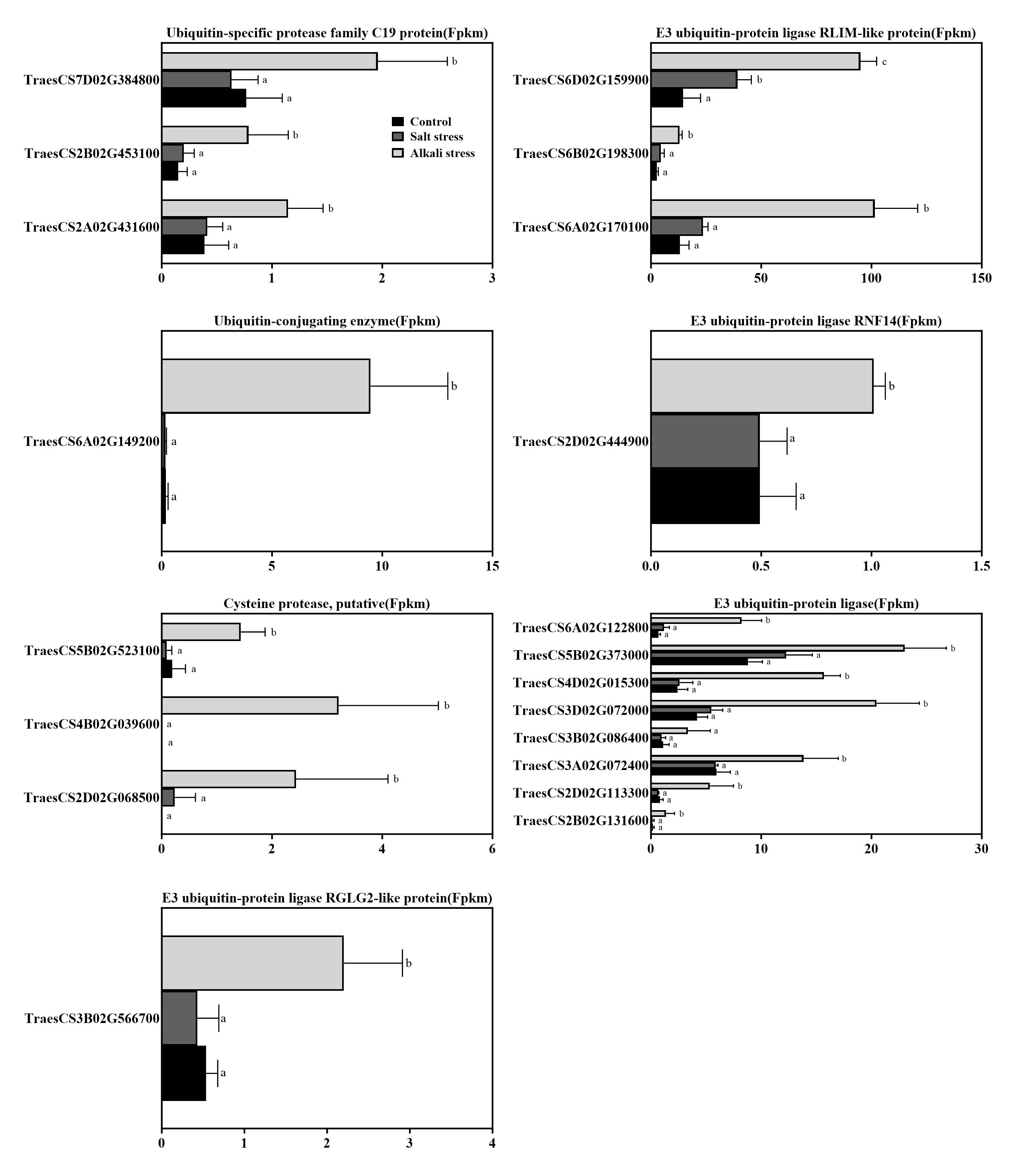

Supplement: Supplementary file 1 [file plants-13-01227-s001.zip › Figure S6.jpg]
